# Classification of chronic pain and spinal cord stimulation response using machine learning in magnetoencephalography data

**DOI:** 10.1371/journal.pone.0337726

**Published:** 2025-12-05

**Authors:** Bart Witjes, Martijn P. A. Starmans, Frank J.P.M. Huygen, Cecile C. de Vos

**Affiliations:** 1 Center for pain Medicine, Department of Anesthesiology, Erasmus University Medical Center, Rotterdam, the Netherlands; 2 Department of Radiology & Nuclear Medicine, Erasmus University Medical Center, Rotterdam, the Netherlands; 3 Department of Pathology, Erasmus University Medical Center, Rotterdam, the Netherlands; FEI: Centro Universitario da FEI, BRAZIL

## Abstract

**Background:**

Due to the complexity of pain, involving physical, psychological, emotional and social aspects, we are still unable to objectively quantify or fully understand this subjective experience. An increasing number of studies have attempted to identify biomarkers of pain using brain imaging tools like magnetoencephalography (MEG). In this study, we used machine learning to investigate the potential of MEG data as a biomarker for chronic pain and used this biomarker to quantify spinal cord stimulation (SCS) treatment effect.

**Methods:**

The study population consisted of 25 patients with SCS, for whom we recorded resting-state MEG during tonic, burst and sham stimulation, 25 patients with chronic pain and 25 pain-free controls. We derived average power spectral densities across each of the 94 automated anatomical labeling atlas based brain regions and extracted six spectral features: the alpha peak frequency, alpha power ratio, and average power across the theta, alpha, beta, and low-gamma bands. Based on these features, we used automated machine learning to find the optimal combination of machine learning methods to create classification and regression models for pain and pain intensity.

**Results:**

The theta power and alpha power ratio were the most promising features to classify chronic pain with an accuracy of 76%. The classification model outputs and self-reported pain scores of patients with SCS showed a Spearman correlation coefficient of 0.12. A regression model based on pain scores of all participants showed Spearman correlation coefficients between 0.27 and 0.41.

**Conclusion:**

This study achieved a promising 76% accuracy in classifying patients with chronic pain and pain-free controls using the theta power or alpha power ratio. However, this model’s output poorly correlated with pain scores of patients with SCS. A larger variety of input features and outcome parameters is recommended.

## Introduction

Chronic pain is pain that persists or recurs for longer than 3 months, affecting approximately 20% of the global population [1]. Chronic pain is a complex condition, involving biological, psychological and social factors. Unlike acute pain, which serves as a warning sign of injury or illness, chronic pain contains little evolutionary benefit and can be considered a disease [2]. It is often characterized by a continuous or intermittent sensation of discomfort, and it can have a significant impact on a person’s daily functioning, mobility, emotional well-being, and overall quality of life.

Due to the complexity of pain, involving physical, psychological, emotional and social aspects, we are still unable to objectively quantify, or fully understand this subjective experience. Therefore, an increasing number of studies have attempted to identify biomarkers of pain [2,3]. These biomarkers could complement self-reports [4] and potentially enhance our understanding of pain, differentiate its origins, or predict treatment efficacy [3,5]. A broad range of measures and assays are currently being studied for their potential as a biomarker of pain and chronic pain. These encompass omics-based biomarkers such as genomics and epigenomics, behavioral analyses such as quantitative sensory testing, imaging techniques such as functional magnetic resonance imaging (fMRI), and electrophysiological assessments such as electroencephalography (EEG) or magnetencephalography (MEG) [5,6]. Using EEG and MEG, the neuronal activity is measured directly. The advantage of using MEG over EEG is that the magnetic fields, unlike the electric fields, generated by the activity of neurons are not distorted by scalp, skull, and cerebrospinal fluid and therefore MEG has a better spatial resolution.

As the search for biomarkers is complex, machine learning can help in recognizing patterns within large datasets and identifying which features or variables are most relevant for predicting pain [3,5,6]. In the current study, we investigated a possible MEG biomarker of chronic pain and its potential to classify treatment effect of spinal cord stimulation (SCS). SCS is a treatment option for patients with refractory chronic pain syndromes like persistent spinal pain syndrome type 2 [7], painful diabetic neuropathy [8] or complex regional pain syndrome [9]. Although SCS is an effective therapy for many patients, the mechanisms of action of SCS are not yet fully understood. In addition, there are currently various stimulation paradigms available and while some patients respond favorably to all paradigms, other patients respond only to specific paradigms or none of the currently available paradigms.

In this study, we aimed to further identify differences in resting-state brain signals between patients with chronic pain and pain-free controls as well as differences in cortical effects of the different treatment responses in patients with SCS. This is the first study to explore the use of a MEG based biomarker of chronic pain to classify SCS treatment effect.

## Methods

**Fig 1:**
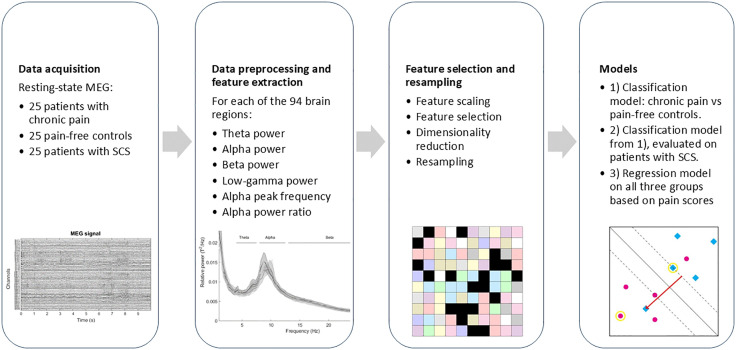
A schematic overview of the methods. Five minutes of resting-state MEG data was recorded from subjects without pain, subjects with chronic pain, and subjects treated with spinal cord stimulation (SCS) who had three recording sessions. After preprocessing of the data, Brainstorm [10] was used to obtain the average time series of 94 brain sources, of which six spectral features were extracted. Subsequently, the WORC toolbox [11,12] was used to optimize feature selection and create three machine learning models.

A schematic overview of the methods is shown in Fig 1.

### Data acquisition

For this study, we used the resting-state MEG data collected between 22/03/2018 and 02/12/2019 for two previous studies [13,14]. Ethics approval was obtained from the institutional review boards of the Montreal Neurological Institute (Montreal, Canada) and the Donders Institute for Brain, Cognition and Behavior (Nijmegen, the Netherlands); all participants provided written informed consent to participate in the study. In the first study [13], we recorded MEG data in patients with chronic pain and healthy controls and found increased slow-to-fast alpha power ratios in patients with chronic pain. In the second study [14], we used MEG to investigate differences in cortical activity in patients with different SCS paradigms, using spectral features: alpha peak frequency, alpha power ratio, and power in theta, alpha, beta and low-gamma band. The study population consisted of 25 patients with SCS, for whom we recorded resting-state brain activity during tonic, burst and sham stimulation, but we did not find differences in the selected spectral features between SCS paradigms.

The study population of our first study [13] consisted of pain-free controls (n = 25) and patients with persistent spinal pain syndrome type 2 (n = 21). In the current study, we added 4 patients with chronic pain with a different etiology (diabetic neuropathy or complex regional pain syndrome) for which SCS is a treatment option (and represented in our patients with SCS), resulting in a study population of 25 patients with chronic pain, 25 pain-free controls and 25 patients with SCS. The participants were age- and sex-matched. The patients with chronic pain as well as the patients with SCS all had pain in their lower body, and patients with SCS had received SCS therapy for at least 6 months. Each participant was asked to fill in standard questionnaires concerning their pain, including the numeric rating scale (NRS) pain score, generic health status (EuroQol 5 dimensions 5 levels), and anxiety and depression (the Hospital Anxiety and Depression Scale [HADS]). We report the EQ5D data using EQ5D index values, with the crosswalk value set from the relevant country (the Netherlands or Canada).

The collected data consist of 5 minute resting-state MEG recordings, conducted with identical setups at the Montreal Neurological Institute (McGill University, Montreal, Canada) or the Donders Institute for Brain, Cognition and Behavior (Nijmegen, the Netherlands). The participants were seated upright under the 275-channel whole-head MEG system (CTF, Coquitlam, BC, Canada) inside a passive magnetically shielded room. The data was recorded with a sampling rate of 2400 Hz with a built-in antialiasing lowpass filter with a 600 Hz cutoff. Electrooculogram and electrocardiogram electrodes captured reference signals for ocular and cardiac artifacts. We used a three-dimensional digitizer system (Polhemus Isotrak) to digitize the participant’s head shape, the respective locations of the head-positioning coils, and anatomical landmarks. Prior to each session, we conducted a 2 min empty-room recording to capture environmental noise to inform the MEG source modeling process [15]. During the recording, the participants were instructed to sit still with their eyes open, maintaining their gaze on a fixation cross. The data acquisition has been described in more detail in previous studies [13,14].

### Data preprocessing and feature extraction

All data processing was performed with Brainstorm using MATLAB version R2020a (The MathWorks, Natick, Massachusetts, USA) [16]. Brainstorm is an open-source app freely available under the GNU general public license [10]. We followed the recommended processing pipeline for MEG preprocessing in Brainstorm [17,18]. Which means that recordings were visually inspected and cleaned from artifacts. Sensors with a low signal-to-noise ratio were excluded from further analysis (varying between 0 and 7 per participant). Notch filters were used to attenuate the powerline’s frequencies and harmonics both for the Netherlands (50, 100, and 150 Hz) and Canada (60, 120, and 180 Hz). A bandpass filter (1–200 Hz) was applied to remove noise. Cardiac and eye-blinking artifacts were attenuated with specific signal-space projections and segments with other types of visible artifacts were excluded from further analysis.

We used Brainstorm to perform MEG source imaging and obtain average MEG source time series at each brain region of the automated anatomical labeling atlas. We derived the average power spectral densities for each of the 94 brain regions and extracted six spectral features from each of these power spectral densities: the alpha peak frequency; the alpha power ratio (power in the 7–9 Hz band divided by the power in the 9–11 Hz band) [13]; and the average power across the theta (4–7.5 Hz), alpha (8–12.5 Hz), beta (13–30 Hz), and low-gamma (30.5–60 Hz) frequency bands. The alpha peak frequency was defined as the frequency with maximum power in the 7–13 Hz range. The exact procedure of the computation of these features is described in our previous papers [13,14].

### Feature selection and machine learning

We used the WORC toolbox (version 3.6.0) in Python (version 3.6.8) to create classification models for pain and pain intensity [11,12]. In WORC, the decision model creation consists of several steps, including feature selection, resampling, and machine learning. WORC performs an automated search amongst a variety of algorithms and hyperparameters for each step, comparing 1000 different combinations, and determines which combination of algorithms and parameters maximizes the classification performance on the training set. The performance of the 100 best performing methods is then averaged into a single decision model (ensemble learning). Compared to manual tuning, this process is quick, easy, objective, automated, reproducible, evaluate a large number of potential methods, and limits method overfitting while improving performance [11,12]. All default settings were used.

First, feature scaling using z-scores was used to avoid the methods using large values only. Next, the MEG spectral features that we used as the input for the model underwent WORC’s default feature selection and dimensionality reduction process. The feature selection process potentially involved various methods: a variance threshold, eliminating features with a low variance (default in WORC: < 0.01); a statistical test procedure (Mann-Whitney U), retaining features with p-values between thresholds; and the use of Relief, retaining features which are important for differentiating between classes. Subsequently, the features potentially underwent principal components analysis, retaining linear combinations of features which explained a large part of the variance in features.

The next steps in the process involved WORC’s default resampling techniques [11] and machine learning methods for classification and regression. For resampling, these were random undersampling, random oversampling, near miss resampling, the neighborhood cleaning rule, SMOTE, and ADASYN. For classification, these were support vector machine, random forest, linear regression, linear discriminant analysis, quadratic discriminant analysis and Gaussian Naïve Bayes. For regression, these were support vector regression, random forest regressor, ElasticNet, LASSO, linear regression, and ridge regression.

### Experimental setup

Three different approaches were used for model training and testing: 1) we developed a classification model based on our data from patients with chronic pain and pain-free controls, evaluated in a leave-one-out cross-validation; 2) we trained a classification model based on all our patients with chronic pain and pain-free controls, and tested the model on patients with SCS; and 3) we developed a regression model across all three groups combined based on NRS pain scores, evaluated in a leave-one-out cross-validation. We ran the models 1 and 3 separately for each of the 6 input features (theta for each of the 94 brain regions, alpha for each of the 94 brain regions, etc.) and once with all input features combined (6x94=564 input features). Model 2 was developed using all input features combined. Each patient with SCS was included three times, as patients were recorded with burst, tonic and sham stimulation. These were separate recordings with separately rated NRS pain scores.

In each experiment, as is default in WORC, a (second internal) 5x random-split cross validation was performed on the training dataset, splitting the data in a training set (80%) and a validation set (20%) for model selection and hyperparameter optimization of the relevant classifier (e.g., support vector machine kernel, depth of random forest trees).

### Evaluation

Performance of the binary classification models was assessed using area under the curve (AUC), accuracy, F1-score, precision, sensitivity and specificity for the classification models. The metric used for hyperparameter optimization of the classification models was the F1-score. Furthermore, we explored which brain areas contributed most in the classification model by exploring the p-values from the Mann Whitney U test of the related features considering a significance level of 0.05. The classification scores, ranging from 0 (no pain) to 1 (chronic pain) for the patients with SCS were plotted against the results from the questionnaires (NRS pain scores, pain duration, HADS, EQ5D and medication usage). The correlation between classification scores and each questionnaire outcome was explored by calculating the Spearman correlation coefficient. The performance of the regression model was assessed using the mean squared error and the Spearman correlation coefficient. The metric used for hyperparameter optimization of the regression model was the R^2^ score (coefficient of determination).

## Results

### Participant characteristics

The data used in this study was obtained from 25 patients with SCS, 25 patients with chronic pain and 25 pain-free controls. The participant characteristics are shown in Table 1. For the patients with SCS, the pain score, EQ5D, HADS anxiety and HADS depression scores are separated for each MEG session (after a week of tonic, burst and sham stimulation).

**Table 1:** Participant characteristics.

	Controls (n = 25)	Patients with chronic pain (n = 25)	Patients with SCS (n = 25)
**Age (years)**	49 ± 11	49 ± 10	56 ± 9
**Sex (M/F)**	15/10	11/14	14/11
**Pain duration (years, mean ± sd)**	N/A	9 ± 8	19 ± 12
**Pain score (NRS, mean ± sd)** ** Tonic** ** Burst** ** Sham**	0 ± 0	5.3 ± 2.4	4.4 ± 2.2 4.4 ± 2.5 4.4 ± 2.1
**EQ5D (mean ± sd)** ** Tonic** ** Burst** ** Sham**	959 ± 34	461 ± 250	662 ± 190 695 ± 172 640 ± 220
**HADS-A (mean ± sd)** ** Tonic** ** Burst** ** Sham**	2 ± 2	9 ± 4	4 ± 3 4 ± 3 4 ± 4
**HADS-D (mean ± sd)** ** Tonic** ** Burst** ** Sham**	1 ± 2	9 ± 4	7 ± 3 7 ± 2 7 ± 2
**Medication**			
** Opiods (#)**	0	15 (60%)	14 (56%)
** Neurotropic drugs (#)**	2 (8%)	18 (72%)	12 (48%)
** NSAID (#)**	1 (4%)	7 (28%)	4 (16%)

SCS; Spinal Cord Stimulation, NRS; Numeric Rating Scale, EQ5D; EuroQol 5 dimensions, HADS-A; Hospital Anxiety and Depression Scale – Anxiety, HADS-D; Hospital Anxiety and Depression Scale – Depression, NSAID; Non-Steroidal Anti-Inflammatory Drug. Age, pain duration, NRS, EQ5D and HADS scores are presented as mean ± standard deviation. Pain score is the NRS score right before the recording.

### Binary classification model and univariate feature analysis

The results for the model for pain classification in patients with chronic pain and pain-free controls are shown in Table 2. The theta frequency showed the highest AUC (0.80), with similar performance to the alpha power ratio (0.75) (Receiver Operating Characteristics in S3 Fig). The only feature group that did not show any predictive value were the low-gamma features with an AUC of 0.51. Combining all features did not yield an improvement in AUC (0.72) over only using the theta frequency or alpha power ratio features.

**Table 2:** Performance for the binary pain classification model that was trained and evaluated on patients with chronic pain and pain-free controls. The performance is shown for the classification with each spectral feature separately, and for the classification of all features combined.

Controls vs Chronic Pain	Theta	Alpha	Beta	Low-Gamma	Peak Frequency	Alpha Power Ratio	Combined
AUC	0.80	0.60	0.58	0.51	0.69	0.75	0.72
Accuracy	0.76	0.56	0.54	0.48	0.70	0.76	0.74
F1-Score	0.76	0.56	0.54	0.48	0.70	0.76	0.74
Precision	0.74	0.55	0.54	0.48	0.71	0.74	0.75
Sensitivity	0.80	0.64	0.52	0.48	0.68	0.80	0.72
Specificity	0.72	0.48	0.56	0.48	0.72	0.72	0.76

AUC; Area under the curve

The Mann Whitney U test showed p-values below 0.05 in 23 brain regions for the theta power, 43 brain regions for the alpha power, none for the beta power, 15 brain regions for the low-gamma power, 38 brain regions for the peak frequency and all 94 brain regions for the alpha power ratio. The regions showing the lowest median of p-values over all features were the temporal inferior gyrus, posterior orbital gyrus, posterior cingulate gyrus and the orbital part of the inferior frontal gyrus. A full overview of the p-values per feature and brain areas is shown in S1 Table.

**Fig 2:**
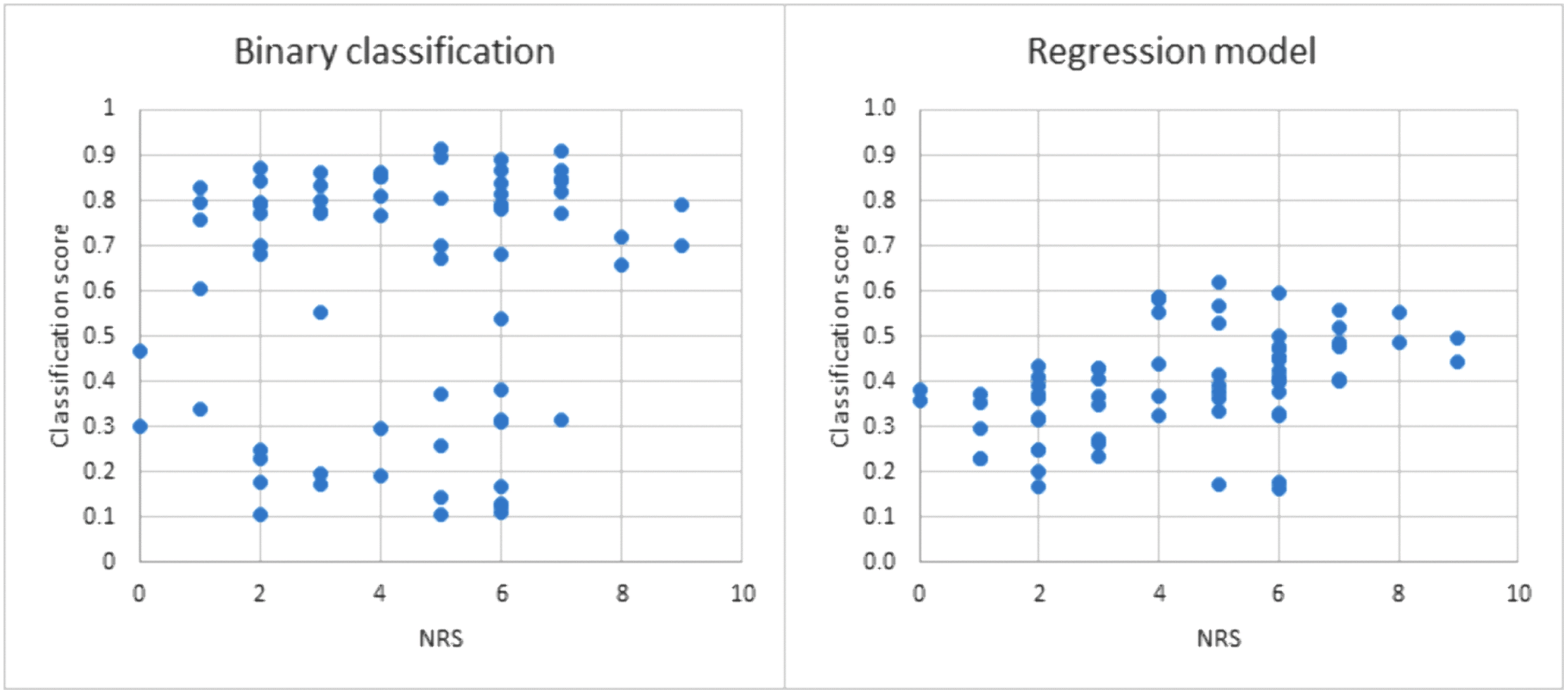
Correlation of classifications against pain scores. **Left**; the classification scores plotted against the numeric rating scale (NRS) pain scores for the binary model with all features combined. The classification scores are plotted for each of the patients with spinal cord stimulation using the model that was trained on patients with chronic pain and pain-free controls. The classification scores and NRS pain scores showed a Spearman correlation coefficient of 0.12. **Right**; the classification scores plotted against the NRS pain scores for the regression model with all features combined. The classification scores are plotted for each patient in all three groups. The classification scores and NRS pain scores showed a Spearman correlation coefficient of 0.41.

The classification scores of the second model for pain classification, which was trained on all features combined and all the data from patients with chronic pain and pain-free controls and tested on the data from patients with SCS, is shown in Fig 2. The classification scores for the patients with SCS, ranging from 0 (no pain) to 1 (pain), and the NRS pain scores showed a Spearman correlation coefficient of 0.12. We also plotted the results from the questionnaires versus the classification scores, but there was no clear relation between these parameters (S2 Fig). The Spearman correlation coefficients of classification score and these questionnaire results were: age 0.16, pain duration 0.21, HADS anxiety 0.24, HADS depression 0.27 and EQ5D score 0.29.

### Regression model

The regression model evaluated in cross-validation on all groups combined (patients with chronic pain, patients with SCS, and pain-free controls) showed Spearman correlation coefficients between 0.27 and 0.41 (Fig 2, Table 3). *The* highest correlation coefficient was acquired using the combination of all spectral features. The mean squared error was between 6.78 and 7.52, indicating that the estimated pain score on average deviated 2.6 from the actual patient reported pain score. The lowest mean squared error was found for the spectral features of the alpha band (6.78).

**Table 3:** Performance of the regression models on patients with chronic pain, patients with spinal cord stimulation and pain-free controls. The models were trained using pain scores ranging from 0 (no pain) to 10 (worst pain imaginable).

Controls, Chronic pain & SCS	Theta	Alpha	Beta	Low-Gamma	Peak Frequency	Alpha Power Ratio	Combined
Mean squared error	7.16	6.78	7.52	7.16	7.09	7.04	6.99
Spearman coefficient	0.34	0.38	0.27	0.35	0.35	0.35	0.41

## Discussion

### Main findings

Our models for binary classification of patients with chronic pain and pain-free controls using either features from the theta frequency power or the alpha power ratio both achieved a promising 76% accuracy. However, the evaluation of the model with all features combined on the patients with SCS did not show a correlation with NRS pain scores, neither did a regression model evaluated on the combined datasets.

### Classification of pain

We are the first study to evaluate a machine learning based MEG biomarker for pain in patients with SCS. Two other studies [19,20] used machine learning to investigate a MEG based biomarker of chronic migraine, with high accuracy (>86.8 and >92.6%) for distinguishing patients with chronic migraine (n = 100) from healthy controls (n = 70), using a support vector machine [19,20]. In the current study, we did not find clear patterns in the used brain areas for classification. Although several regions showing low p-values are known to be involved in pain, such as the prefrontal and cingulate cortices, the area showing the lowest median p-value, the temporal inferior gyrus, is not specifically known for the processing of pain. Our study shows additional brain regions could help distinguishing pain from no pain. Comparing our study to the above [19,20], adding connectivity measures to our model like the phase locking value or phase lag index between the regions with low p-values (S1 Table) could potentially improve performance in future research.

Alternatively to MEG, an increasing number of studies have used machine learning and EEG with the objective to classify chronic pain [3,6]. The accuracy of the studies classifying chronic pain ranges between 55% and 93% [3]. There is a large variety in studied pain syndromes, EEG features studied, machine learning methods, and model performance [6]. While EEG involves lower costs and is more available, the magnetic fields recorded by MEG are not distorted by scalp, skull, and cerebrospinal fluid, resulting in a better spatial resolution in source localization. The better spatial resolution could help improving model outcomes if activity in distinct brain regions are unique for specific pain syndromes. This was also demonstrated by Hsiao et al. who showed accuracy >75.7% in discriminating patients with chronic migraine from patients with fibromyalgia, chronic tension-type headache or episodic migraine on MEG [19,20]. Due to the large variety in study designs, it is hard to directly compare the EEG studies with the MEG studies. Therefore, it is yet unclear which of these two techniques is most suitable as chronic pain biomarker, and further research directly comparing and potentially combining EEG and MEG biomarkers is required.

### Classification of SCS treatment effect

The classification scores of the patients with SCS correlated with none of the clinical variables available in this study (NRS pain scores, pain duration, anxiety scores, depression score, EQ5D, and medication usage). However, the model was trained to distinguish patients with chronic pain from pain-free controls, but also patients with successful SCS (>50% reduction in NRS pain score) often still experience some pain. To explore the possibility to estimate pain scores instead of binary classification (pain vs no pain), we also developed a regression model based on the NRS pain scores to classify treatment effect. However, the regression model also showed poor performance.

Looking at the classification scores plotted against the NRS pain scores in the patients with SCS (Fig 2), there seem to be two groups, as most patients showed a classification score either below 0.4 or above 0.6. We could not relate the classification scores nor these groups to NRS pain scores or any of the other questionnaire outcomes (S2 Fig). However, pain scores are subjective and are difficult to compare over subjects. Our biomarker indicates objective differences in the MEG data, and therefore the classification scores could still indicate successful versus unsuccessful SCS as a change in MEG data rather than a change in pain scores. Since the model created two groups, but the two groups did not correlate with any of the questionnaire outcomes, unsupervised learning (e.g., cluster analysis) could be useful for future research. This opens up the possibility to classify groups in the MEG data without needing to provide ground truth labels such as subjective pain scores. A relatively straightforward option is to use the same method as in our study, only replacing the classification or regression algorithms with unsupervised clustering algorithms. A challenge in clustering analysis is defining the number of clusters and post-analysis finding patterns in the defined clusters, as the method is unsupervised. In this case, logical clusters could be potentially based on pain (e.g., three clusters: no pain (=controls), light pain requiring no treatment, high pain requiring treatment), or treatment response (e.g., no response, some response, good response), motivating the choice for a low (around three) number of clusters and analyzing whether these patterns are present. As pain is a multidimensional experience, ideally, a broader range of variables should be taken into account to capture the holistic treatment response of SCS [21]. Since our MEG-based machine learning model indicated different groups within the SCS treated patients, this could potentially be used as one of the factors.

### Limitations

A limitation of our study is the relatively small sample size compared to the aforementioned studies [19,20]. Larger sample sizes could increase performance and would lead to more reliable and consistent performance across various conditions and datasets. In addition, validating our model’s performance with an independent data set could have been valuable, though it would have further reduced our sample size. To prevent overfitting on a relatively small dataset, we used WORC’s extensive portfolio of feature selection techniques, and a rigorous evaluation setup with cross-validation to perform any model optimization on the training dataset. These have previously proven successful on smaller datasets with a higher number of features to samples as in our study [11]. Second, we used a limited amount of resting-state features. Adding a larger variety of resting-state features, such as connectivity measures such as mentioned above, could improve performance further. Third, we used data from patients with various SCS paradigms to include a variety of pain intensities, but this might have complicated the interpretation of the data because of intertwined effects of SCS itself and the pain relief it caused. Lastly, it is important to note that confounding factors might have influenced our results. Examples of such factors include medication usage or psychologic factors such as anxiety and depression, which are both more prevalent in subjects experiencing chronic pain. However, we did not find strong correlations of these factors with the model’s classification scores.

### Future research

First, as mentioned above, adding more subjects, preferably from multiple centers, and a larger variety of resting-state features, and MEG or EEG features from study designs using evoked potentials or conditioned pain modulation could further optimize classification. Second, clinical parameters such as vital signs, medical history, etc. could be considered as input features for classification [22,23]. Third, baseline (pre-implantation) information of the patients with SCS was not available for this study. Treatment responses could therefore only be evaluated using questionnaire results at the time of the MEG recordings. Classification models based on changes in MEG activity as well as perceived pain compared to pre-implantation baseline may provide additional insights. More information on the most relevant MEG spectral features might then be obtained by using model explainability techniques. Combined with cluster analysis, this might indicate multiple groups (e.g., tonic SCS responder, tonic SCS non-responder, burst SCS responder, etc.). Lastly, as we expand the sample size and encompass a wider range of studied parameters, the implementation of deep learning techniques could contribute to improving classification performance similar to related EEG studies [3,6].

## Conclusion

Our machine learning-based model achieved a promising 76% accuracy in classifying patients with chronic pain and pain-free controls based on the MEG theta frequency power or the alpha power ratio. However, the model output poorly correlated with pain scores of patients with SCS. For future studies, we recommend using a larger variety of features and training the classification model on a larger variety of outcome parameters instead of pain scores alone, as many factors are involved in chronic pain and SCS treatment effectiveness.

## Supporting information

S1 Table The p-values per feature and per brain region from the binary classification model (patients with pain and pain-free controls), computed using the Mann-Whitney U test. The values are sorted based on the median of the p-value of all features. The brain regions are labeled using the automated anatomical labelling atlas.
(DOCX)

S2 Fig The classification scores of the regression model trained using the numeric rating scale pain scores. The classification scores range from 0 (no pain) to 1 (chronic pain). The Spearman correlation coefficients of classification score and questionnaire results were: age 0.16, pain duration 0.21, HADS anxiety 0.24, HADS depression 0.27 and EQ5D score 0.29.
(DOCX)

S3 Fig Receiver operating characteristics for the classification model using theta features (left) and the slow to fast alpha power ratio features (right). (DOCX)

S4 Text PLOS’ Inclusivity in global research questionnaire. (DOCX)
