# Supplementary material for: Classification of chronic pain and spinal cord stimulation response using machine learning in magnetoencephalography data
Source: PLoS One. 2025 Dec 5;20(12):e0337726. doi: 10.1371/journal.pone.0337726 (PMC12680202; doi:10.1371/journal.pone.0337726)
Supplement: S1 Table — The values are sorted based on the median of the p-value of all features. The brain regions are labeled using the automated anatomical labelling atlas. (DOCX) [file pone.0337726.s001.docx]

## S1 The p-values per feature per brain region

S1 Table: The p-values per feature and per brain region from the binary classification model (patients with pain and pain-free controls), computed using the Mann-Whitney U test. The values are sorted based on the median of the p-value of all features. The brain regions are labeled using the automated anatomical labelling atlas.

| **Brain region** | **Theta** | **Alpha** | **Beta** | **Low-gamma** | **Peak frequency** | **Alpha power ratio** | **Median of p-values** |
| --- | --- | --- | --- | --- | --- | --- | --- |
| Temporal_Inf_R | 0.0010 | 0.1565 | 0.1430 | 0.0199 | 0.0260 | 0.0003 | 0.0229 |
| OFCpost_L | 0.4460 | 0.0274 | 0.1913 | 0.0341 | 0.0004 | 0.0026 | 0.0307 |
| Cingulate_Post_L | 0.0250 | 0.0341 | 0.2547 | 0.4383 | 0.0315 | 0.0012 | 0.0328 |
| Frontal_Inf_Orb_2_R | 0.2803 | 0.0457 | 0.1261 | 0.0239 | 0.0020 | 0.0017 | 0.0348 |
| Olfactory_R | 0.0439 | 0.1783 | 0.2076 | 0.0286 | 0.0226 | 0.0020 | 0.0362 |
| Temporal_Pole_Mid_R | 0.0457 | 0.1430 | 0.2076 | 0.0181 | 0.0330 | 0.0028 | 0.0394 |
| Temporal_Pole_Sup_L | 0.4231 | 0.0356 | 0.1036 | 0.0476 | 0.0008 | 0.0008 | 0.0416 |
| Cingulate_Post_R | 0.0172 | 0.0476 | 0.2803 | 0.4923 | 0.0367 | 0.0007 | 0.0422 |
| Precuneus_R | 0.0110 | 0.0239 | 0.3070 | 0.3418 | 0.0619 | 0.0003 | 0.0429 |
| Olfactory_L | 0.1302 | 0.0728 | 0.2188 | 0.0156 | 0.0062 | 0.0012 | 0.0442 |
| Amygdala_L | 0.1344 | 0.0457 | 0.1612 | 0.0439 | 0.0015 | 0.0010 | 0.0448 |
| Hippocampus_R | 0.0341 | 0.0580 | 0.1222 | 0.0558 | 0.0056 | 0.0001 | 0.0449 |
| Temporal_Pole_Sup_R | 0.0603 | 0.1072 | 0.2020 | 0.0326 | 0.0170 | 0.0033 | 0.0465 |
| Insula_L | 0.4079 | 0.0208 | 0.1758 | 0.0755 | 0.0026 | 0.0007 | 0.0482 |
| OFCant_R | 0.2868 | 0.0099 | 0.1809 | 0.0812 | 0.0175 | 0.0049 | 0.0493 |
| OFCant_L | 0.3708 | 0.0239 | 0.3277 | 0.0755 | 0.0220 | 0.0023 | 0.0497 |
| ParaHippocampal_R | 0.0208 | 0.1108 | 0.2738 | 0.0812 | 0.0169 | 0.0003 | 0.0510 |
| Putamen_L | 0.3138 | 0.0371 | 0.2076 | 0.0651 | 0.0032 | 0.0006 | 0.0511 |
| Fusiform_R | 0.0099 | 0.1302 | 0.4460 | 0.0968 | 0.0201 | 0.0005 | 0.0585 |
| Angular_R | 0.0558 | 0.0457 | 0.2246 | 0.2516 | 0.0644 | 0.0003 | 0.0601 |
| Temporal_Pole_Mid_L | 0.4155 | 0.0627 | 0.1430 | 0.0580 | 0.0027 | 0.0018 | 0.0603 |
| Pallidum_L | 0.1758 | 0.0516 | 0.2132 | 0.0702 | 0.0178 | 0.0006 | 0.0609 |
| Putamen_R | 0.0676 | 0.0558 | 0.3418 | 0.1090 | 0.0236 | 0.0006 | 0.0617 |
| Amygdala_R | 0.0326 | 0.0935 | 0.1612 | 0.0356 | 0.0962 | 0.0025 | 0.0645 |
| Temporal_Mid_R | 0.0341 | 0.0603 | 0.3490 | 0.0702 | 0.1101 | 0.0003 | 0.0652 |
| Occipital_Inf_R | 0.0164 | 0.2424 | 0.1660 | 0.1072 | 0.0302 | 0.0007 | 0.0687 |
| Caudate_L | 0.1262 | 0.0387 | 0.3855 | 0.0903 | 0.0471 | 0.0003 | 0.0687 |
| Frontal_Sup_Medial_R | 0.1660 | 0.0356 | 0.0728 | 0.0663 | 0.2863 | 0.0274 | 0.0696 |
| Frontal_Inf_Tri_R | 0.3635 | 0.0476 | 0.2803 | 0.0812 | 0.0581 | 0.0004 | 0.0696 |
| OFCpost_R | 0.1913 | 0.1183 | 0.1262 | 0.0250 | 0.0142 | 0.0039 | 0.0717 |
| OFClat_R | 0.1386 | 0.0812 | 0.0903 | 0.0239 | 0.0629 | 0.0049 | 0.0721 |
| Pallidum_R | 0.0603 | 0.0603 | 0.3347 | 0.1344 | 0.0850 | 0.0008 | 0.0727 |
| OFCmed_L | 0.3781 | 0.0903 | 0.3277 | 0.0286 | 0.0714 | 0.0026 | 0.0808 |
| Caudate_R | 0.0702 | 0.0935 | 0.4383 | 0.1002 | 0.0671 | 0.0015 | 0.0818 |
| Cuneus_L | 0.0218 | 0.0968 | 0.2803 | 0.3855 | 0.0718 | 0.0042 | 0.0843 |
| Frontal_Inf_Orb_2_L | 0.2485 | 0.0149 | 0.1612 | 0.1861 | 0.0031 | 0.0047 | 0.0880 |
| Lingual_R | 0.0172 | 0.2020 | 0.1612 | 0.2305 | 0.0108 | 0.0003 | 0.0892 |
| Thalamus_R | 0.1262 | 0.0537 | 0.3138 | 0.1474 | 0.0103 | 0.0004 | 0.0899 |
| Insula_R | 0.1222 | 0.0580 | 0.4004 | 0.1612 | 0.0416 | 0.0005 | 0.0901 |
| Frontal_Sup_Medial_L | 0.2076 | 0.0274 | 0.0728 | 0.1108 | 0.2506 | 0.0181 | 0.0918 |
| Temporal_Inf_L | 0.1809 | 0.1612 | 0.1344 | 0.0558 | 0.0330 | 0.0026 | 0.0951 |
| Calcarine_L | 0.0149 | 0.1386 | 0.2076 | 0.4460 | 0.0571 | 0.0025 | 0.0979 |
| Rectus_L | 0.2547 | 0.1222 | 0.3635 | 0.0476 | 0.0738 | 0.0028 | 0.0980 |
| Calcarine_R | 0.0128 | 0.1002 | 0.2803 | 0.3418 | 0.0961 | 0.0002 | 0.0981 |
| Occipital_Sup_R | 0.0457 | 0.0812 | 0.4691 | 0.2610 | 0.1154 | 0.0005 | 0.0983 |
| Lingual_L | 0.0476 | 0.2188 | 0.1565 | 0.2803 | 0.0307 | 0.0009 | 0.1020 |
| Cingulate_Mid_R | 0.1302 | 0.0326 | 0.2132 | 0.4307 | 0.0750 | 0.0013 | 0.1026 |
| Heschl_L | 0.5000 | 0.0356 | 0.1758 | 0.3929 | 0.0177 | 0.0028 | 0.1057 |
| OFClat_L | 0.2868 | 0.0105 | 0.3207 | 0.2020 | 0.0042 | 0.0072 | 0.1063 |
| Temporal_Sup_R | 0.0476 | 0.0239 | 0.2935 | 0.1660 | 0.2069 | 0.0002 | 0.1068 |
| Temporal_Mid_L | 0.2132 | 0.0580 | 0.2305 | 0.1565 | 0.0434 | 0.0017 | 0.1073 |
| Heschl_R | 0.0537 | 0.0404 | 0.3708 | 0.1660 | 0.2511 | 0.0001 | 0.1098 |
| Parietal_Inf_R | 0.1474 | 0.0728 | 0.3490 | 0.4691 | 0.0401 | 0.0012 | 0.1101 |
| Cuneus_R | 0.0116 | 0.0651 | 0.3138 | 0.4231 | 0.1556 | 0.0005 | 0.1103 |
| Cingulate_Mid_L | 0.0935 | 0.0313 | 0.3207 | 0.4383 | 0.1294 | 0.0012 | 0.1115 |
| Frontal_Inf_Oper_R | 0.1758 | 0.1108 | 0.3347 | 0.1145 | 0.0392 | 0.0009 | 0.1126 |
| Rolandic_Oper_R | 0.1145 | 0.0627 | 0.4691 | 0.1709 | 0.1120 | 0.0002 | 0.1133 |
| Hippocampus_L | 0.2673 | 0.0872 | 0.2935 | 0.1519 | 0.0645 | 0.0017 | 0.1195 |
| Occipital_Mid_R | 0.1145 | 0.0935 | 0.4004 | 0.1519 | 0.1315 | 0.0006 | 0.1230 |
| ParaHippocampal_L | 0.2246 | 0.1222 | 0.4537 | 0.1302 | 0.0668 | 0.0012 | 0.1262 |
| Parietal_Sup_L | 0.0872 | 0.0421 | 0.4155 | 0.4231 | 0.1701 | 0.0016 | 0.1287 |
| Precuneus_L | 0.0387 | 0.0164 | 0.2868 | 0.2246 | 0.4535 | 0.0013 | 0.1317 |
| Thalamus_L | 0.3002 | 0.0421 | 0.3347 | 0.2246 | 0.0322 | 0.0039 | 0.1334 |
| Frontal_Sup_2_L | 0.2132 | 0.0164 | 0.1302 | 0.2547 | 0.1387 | 0.0058 | 0.1345 |
| Parietal_Inf_L | 0.1913 | 0.0421 | 0.4614 | 0.4537 | 0.0808 | 0.0025 | 0.1360 |
| SupraMarginal_R | 0.0872 | 0.1002 | 0.4004 | 0.1758 | 0.4040 | 0.0002 | 0.1380 |
| Frontal_Mid_2_L | 0.1861 | 0.0356 | 0.0903 | 0.2246 | 0.2016 | 0.0049 | 0.1382 |
| Rolandic_Oper_L | 0.5000 | 0.0172 | 0.2485 | 0.4231 | 0.0338 | 0.0018 | 0.1412 |
| Occipital_Inf_L | 0.0676 | 0.3207 | 0.2424 | 0.1913 | 0.0978 | 0.0023 | 0.1446 |
| Occipital_Sup_L | 0.0313 | 0.0935 | 0.3347 | 0.3855 | 0.2094 | 0.0069 | 0.1515 |
| Frontal_Inf_Oper_L | 0.4383 | 0.0421 | 0.1108 | 0.1966 | 0.4191 | 0.0164 | 0.1537 |
| Frontal_Inf_Tri_L | 0.2076 | 0.0128 | 0.1036 | 0.2610 | 0.3961 | 0.0116 | 0.1556 |
| Frontal_Med_Orb_R | 0.4383 | 0.1072 | 0.2076 | 0.0603 | 0.4411 | 0.0164 | 0.1574 |
| Frontal_Mid_2_R | 0.4383 | 0.1036 | 0.2132 | 0.0676 | 0.3541 | 0.0072 | 0.1584 |
| Fusiform_L | 0.1474 | 0.2020 | 0.4079 | 0.1145 | 0.1850 | 0.0018 | 0.1662 |
| Frontal_Med_Orb_L | 0.4923 | 0.1262 | 0.2305 | 0.1036 | 0.2111 | 0.0062 | 0.1686 |
| Postcentral_R | 0.2485 | 0.0903 | 0.4231 | 0.2738 | 0.0564 | 0.0003 | 0.1694 |
| Parietal_Sup_R | 0.1344 | 0.0356 | 0.4307 | 0.3347 | 0.2239 | 0.0016 | 0.1791 |
| OFCmed_R | 0.3347 | 0.1145 | 0.2485 | 0.0526 | 0.2599 | 0.0085 | 0.1815 |
| Occipital_Mid_L | 0.0421 | 0.0842 | 0.4307 | 0.3347 | 0.2964 | 0.0029 | 0.1903 |
| Rectus_R | 0.1993 | 0.1966 | 0.3490 | 0.0439 | 0.1851 | 0.0044 | 0.1909 |
| Angular_L | 0.1183 | 0.0250 | 0.2738 | 0.4768 | 0.3778 | 0.0033 | 0.1960 |
| Frontal_Sup_2_R | 0.3347 | 0.0404 | 0.2305 | 0.2020 | 0.1970 | 0.0033 | 0.1995 |
| Cingulate_Ant_R | 0.3490 | 0.0439 | 0.2935 | 0.2305 | 0.1738 | 0.0055 | 0.2021 |
| Precentral_L | 0.4460 | 0.0404 | 0.3418 | 0.3929 | 0.0736 | 0.0010 | 0.2077 |
| SupraMarginal_L | 0.2803 | 0.0457 | 0.3070 | 0.4614 | 0.1491 | 0.0085 | 0.2147 |
| Cingulate_Ant_L | 0.4845 | 0.0968 | 0.2485 | 0.2132 | 0.2213 | 0.0033 | 0.2172 |
| Postcentral_L | 0.3562 | 0.0239 | 0.4460 | 0.3635 | 0.0882 | 0.0003 | 0.2222 |
| Supp_Motor_Area_R | 0.3635 | 0.0603 | 0.3708 | 0.4691 | 0.0821 | 0.0055 | 0.2228 |
| Supp_Motor_Area_L | 0.4845 | 0.0379 | 0.4079 | 0.4231 | 0.0213 | 0.0035 | 0.2229 |
| Temporal_Sup_L | 0.4691 | 0.0286 | 0.2246 | 0.2935 | 0.2238 | 0.0047 | 0.2242 |
| Precentral_R | 0.3207 | 0.1565 | 0.4307 | 0.3855 | 0.0881 | 0.0025 | 0.2386 |
| Paracentral_Lobule_R | 0.1861 | 0.0286 | 0.4004 | 0.3490 | 0.4190 | 0.0005 | 0.2675 |
| Paracentral_Lobule_L | 0.2485 | 0.0439 | 0.4307 | 0.4307 | 0.3486 | 0.0015 | 0.2985 |
