# Supplementary material for: Classification of chronic pain and spinal cord stimulation response using machine learning in magnetoencephalography data
Source: PLoS One. 2025 Dec 5;20(12):e0337726. doi: 10.1371/journal.pone.0337726 (PMC12680202; doi:10.1371/journal.pone.0337726)
Supplement: S2 Fig — The classification scores range from 0 (no pain) to 1 (chronic pain). The Spearman correlation coefficients of classification score and questionnaire results were: age 0.16, pain duration 0.21, HADS anxiety 0.24, HADS depression 0.27 and EQ5D score 0.29. (DOCX) [file pone.0337726.s002.docx]

## S2: Questionnaire results versus the classification scores

S2 Figure: The classification scores of the regression model trained using the numeric rating scale pain scores. The classification scores range from 0 (no pain) to 1 (chronic pain). The Spearman correlation coefficients of classification score and questionnaire results were: age 0.16, pain duration 0.21, HADS anxiety 0.24, HADS depression 0.27 and EQ5D score 0.29.
