# Supplementary material for: Classification of chronic pain and spinal cord stimulation response using machine learning in magnetoencephalography data
Source: PLoS One. 2025 Dec 5;20(12):e0337726. doi: 10.1371/journal.pone.0337726 (PMC12680202; doi:10.1371/journal.pone.0337726)
Supplement: S3 Fig — (DOCX) [file pone.0337726.s003.docx]

## S3: Receiver operating characteristic for Theta and Ratio


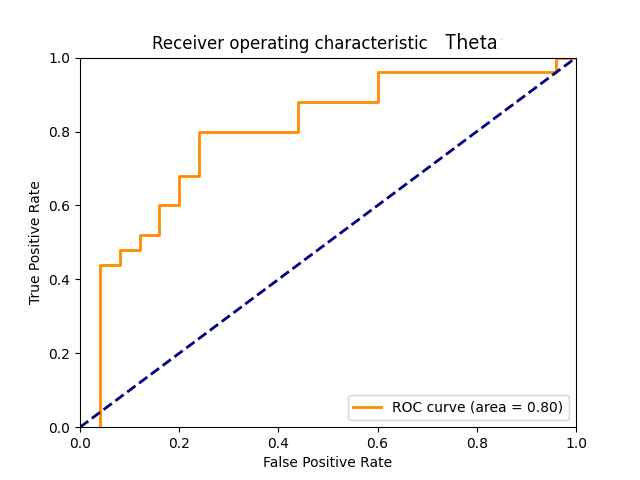

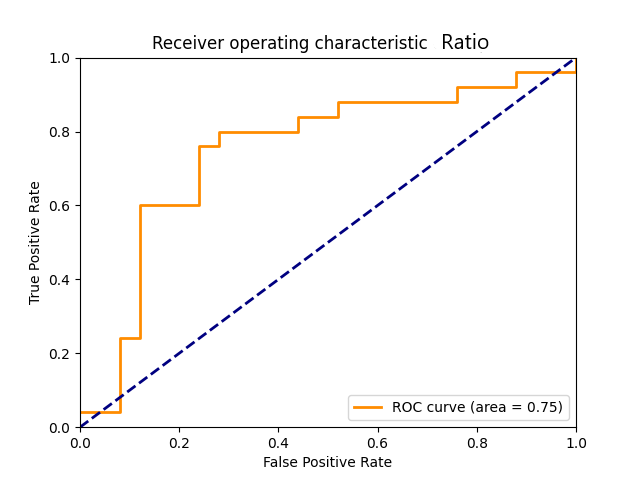


S3 Figure: Receiver operating characteristics for the classification model using theta features (left) and the slow to fast alpha power ratio features (right)
